# Supplementary material for: Trust dynamics among aquaculture producers and regulators in the midwestern United States
Source: PLoS One. 2026 Oct 15;21(10):e0358442. doi: 10.1371/journal.pone.0358442 (PMC13671553; doi:10.1371/journal.pone.0358442)
Supplement: Supplementary file 1 [file pone.0358442.s007.pdf]

Manuscript

Click here to access/download;Manuscript;aquaculture_trust_plos_one.docx

Trust dynamics among aquaculture producers and regulators in the Midwestern United States

Haley Hartenstine, J. Stuart Carlton*

Coastal & Great Lakes Social Science Lab/Illinois-Indiana Sea Grant/Department of Forestry & Natural Resources, Purdue University, West Lafayette, Indiana, United States of America

*Corresponding author

E-mail: carltons@purdue.edu (SC)

Abstract

This qualitative study explores the relationship between producers and regulators in aquaculture production in the midwestern United States. We use the trust ecology framework to illustrate both how different dimensions of trust are present among aquaculture producers and how the dimensions of trust might be threatened. Our findings emphasize the importance of building stronger interpersonal relationships between regulatory agencies and aquaculture producers. Additionally, our research shows that producers feel a need for more vocal advocacy for the industry within the regulatory system. These findings ~~present an important case study~~ illustrating how trust influences the relationship between producers and regulators and how building trust might allow the industry to work together to grow production and, potentially, sustainability.

do not judge your own work!

Introduction

Sustainably meeting future food production needs will require balancing potentially conflicting goals: the desire to "feed the world" for a growing global population and the desire to do so in a way that minimizes the environmental impact of food production and maximizes sustainability (Foley, 2011). This will require a substantial transformation of food production systems, particularly in the United States, to increase environmental sustainability and economic resilience without compromising food availability or security (Traldi et al., 2024).

Such a large-scale transformation will not be easy. It will, among other things, require careful cooperation between food producers and regulators to revise production methods and regulations. Such cooperation demands a sociopolitical environment that permits policy and production experimentation. Trust among producers, regulators, and resource managers has been shown to facilitate such an environment (Lubell 2007). But what builds trust? What destroys it? Can trust, once lost, be repaired? In this research, we examine these and related issues among aquaculture producers and regulators in the US Great Lakes region.

Trust is critical to successful resource management

Trust is a key part of successful resource management. Trust among those who govern and those who are governed is a key part of democratic systems and a key aspect of good governance (Beshi and Kaur 2020). On a more pragmatic level, higher-trust resource management regimes foster support for resource management actions (Carlton et al., 2023; Carlton et al.,2024), build a positive atmosphere of policy experimentation (Westermann, Ashby, and Pretty 2005), and helps to create solutions to collective action policy problems (Jakku et al. 2019; Lubell 2007).

In agriculture, trust is a particularly important form of social capital. To understand why, consider two foundational challenges of farming: the challenge of successfully growing crops at scale in an era of increased weather and climatic variability and the challenge of navigating the complex regulatory and financial landscape of modern agriculture. Managing both challenges successfully is beyond many individuals: the knowledge required is too dense, too esoteric, and too quickly evolving. As a result, most farmers turn

to experts—crop advisors, seed dealers, insurance brokers, cooperative extension staff, etc.—for help (Prokopy et al., 2015).

Working with these sorts of experts creates a relationship with information asymmetry where the experts possess knowledge that the farmers do not (Giampietri et al. 2020). By relying on expert knowledge and opinion, farmers put themselves in a place of vulnerability: their success depends on the quality of the information shared by the experts. Trust is what allows farmers to accept this vulnerability (Stern and Baird 2015) and enables farmers to be open to the advice they need to make decisions in the modern agricultural system. Trust is key for farmers to work with the experts: without it, they are unlikely to receive and act on farm management advice. Research has born this out: trust has been shown to help farmers incorporate climate information into their farm management decisions (Mase et al. 2020; Prokopy et al. 2015), better adopt financial tools to help mitigate risk (Giampietri et al. 2020), and adopt conservation tools and practices (e.g., Lubell et al. 2013).

Although trust is critical in agriculture, it is frequently hard to come by and slow to develop (Rust et al. 2020). Personal characteristics and interpersonal relationships are often key to developing trust in expertise among farmers. For example, farmers are more likely to trust experts they work directly with—such as crop advisors or seed dealers—than those who are farther away—such as university scientists (Prokopy et al., 2015). Reputation matters, as well: farmers often trust experts with a history of working in agriculture or from universities or institutions with a longer history of working in agriculture than scientists perceived as outsiders (Rust et al. 2022). Perceived motives matter, especially when it

comes to regulations: there is often a perception that the state is concerned with “big ag” rather than smaller or non-conventional farming operations (Carolan et al., 2006).

Having established that trust enables farmers to navigate modern agriculture and allows experts ranging from scientists to regulators to successfully interact with farmers, important questions remain: what, exactly, is trust? How can it be defined? How can it be measured?

The trust ecology framework

In this study, we define and measure trust using Stern and Baird’s (2015) trust ecology framework. Stern and Baird define trust as “an individual’s willingness to accept vulnerability in the face of uncertainty” and—building off prior work by Stern and Coleman (2015)—they focus on four dimensions of trust that are relevant to natural resource management: dispositional, rational, affinitive, and procedural trusts. These four dimensions are distinct yet intertwined, and the relative abundance of each can play a key role in understanding trust diversity, and thus resilience, within an institution. Based on Stern and Baird (2015), we define the dimensions as follows:

Dispositional trust describes an individual’s general tendency to trust or distrust. This tendency could be an innate trait of the individual, or based on their life experiences, personal history, or cultural norms. Dispositional trust is the basis from which trust can be built or weakened.

of trusting them. High levels of rational trust allow institutional progress to be made via agreement on actions, information sharing, collaboration, and learning, to name a few. Rational trust can be broken in various ways, such as a performance failure by the trustee that leads the trustor to believe they are less competent or that trusting them will lead to harm, loss, or failure for the trustor.

Affinitive trust focuses on the trustor's judgement of the potential trustee's character and integrity, as well as perceptions of shared values and social connectedness (or lack thereof). It is usually developed or strengthened through positive shared experiences in which the trustee demonstrates having the trustor's best interests at heart, as well as exhibiting responsiveness and active listening to the trustor (Braithwaite 1998; Cvetkovich and Winter 2003; Stern 2008; Stern and Baird 2015). This form of trust can be easily broken or threatened by negative shared experiences between the trustor and trustee, as well as a mismatch in values or perceived lack of integrity of the trustee. Depending on the context, other forms of trust can help to compensate for areas in which there is not strong affinitive trust (Stern and Baird 2015).

Procedural trust, also known as system-based trust, focuses on procedures, systems, or rules that—when perceived as legitimate and when operating correctly—create a safety net for the trustor that promotes trust. This form of trust is generally weakened when the trustors do not perceive the systems in place to be operating fairly or correctly, or when there are constantly changing policies and personnel turnovers that threaten the stability of the systems (Stern and Baird 2015).

There are three forms that trust can take between a trustor and potential trustee: *positive trust* (just referred to as "trust") *distrust*, and *lack of trust* (see Table 2). These forms of trust exist on two separate spectrums, one ranging from lack of trust to positive trust, and the other ranging from lack of trust to distrust. Lack of trust is neither trust nor distrust; it occurs when the trustor does not have enough information about the trustee to determine whether they can or should be trusted. Trust and distrust, on the other hand, involve an explicit expectation or judgement about the trustee, usually drawing upon past experiences (Stern and Baird 2015; Lewicki et al. 2006).

Trust ecology analogizes trust dynamics to ecosystem dynamics: institutions, like ecosystems, can develop resilience to overcome disturbances. Just like high degrees of species richness and evenness lead to ecosystem resilience through functional redundancy, high levels of trust richness and evenness can lead to strong trust diversity within an institution, supporting resilience. With higher trust diversity, a disturbance in one type of trust can be buffered by other types of trust, preserving institutional functions. Conversely, low levels of trust richness and evenness can weaken resilience.

It is important to note that the trust ecology framework only considers positive trust of each type as counting toward its abundance. Distrust and lack of trust do not contribute to diversity, though they are important as they can potentially threaten institutional resilience or serve a positive function by encouraging more stakeholders to actively participate and contribute to NRM. However, Stern and Baird (2015) note that this positive function only occurs when there is at least some degree of system-wide trust as well. Distrust also provides interesting insight into potential trust disturbances and repair strategies.

Trust ecology also considers *trust repair*: the critical steps that need to be taken when trust is broken. After a fracture in trust, people tend to focus more on that which reinforces their distrust rather than any evidence for positive (Slovic 1993; Kim et al. 2004). Therefore, Stern and Baird (2015) suggest that trust repair efforts must aim to simultaneously overcome negative expectations and restore or create positive expectations about future trust relationships. Trust repair may come in several different forms, including *distrust regulation* and *trustworthiness demonstration* (Gillespie and Dietz 2009). Distrust regulation works to prevent future loss of trust by addressing the faults that caused the original breakage. Trustworthiness demonstration involves affinitive trust rebuilding such as apologizing, taking responsibility, and offering reparations, as well as rational components such as setting up ways to display competence. In our case, it will be important to hear from the producers themselves about ways in which they believe their broken trust relationships could be repaired.

The present research

In this research, we use aquaculture in the midwestern United States as a case study examining issues of trust in food production and regulation. Aquaculture is potentially a key part of a sustainable food system because demand for seafood is increasing rapidly but natural fish stocks remain a finite resource (Colyn et al. 2014). Aquaculture will continue to be essential in feeding the billions of people in the world who depend on seafood as an important source of protein, and expansion of the industry can help to ensure future global food security (Beveridge et al., 2010). Additionally, it can help offset the demand on wild

fish stocks, aid in restoration of habitat for at-risk species, and contribute to economic growth in local communities (National Marine Fisheries Service (NMFS) 2021).

Despite seafood's importance and popularity, the US aquaculture industry is relatively small. The US currently ranks 17th in global aquaculture production (NMFS 2021). Although aquaculture has been the food production technology with the fastest growth rate for several decades, the US has struggled to see growth in the domestic aquaculture industry, and in some years even experienced negative growth rates in aquaculture production (Garlock et al. 2020). As a result, between 70 and 85 percent of the seafood consumed in the US is imported, and about 50 percent of this imported seafood is farmed (NMFS 2021). As of 2019, the US seafood trade deficit had grown to $16.9 billion and it is predicted to continue rising (NMFS 2021; United States Department of Agriculture 2019).

Given the substantial coastal and inland water resources available to raise seafood, the US has vast unrealized potential for aquaculture growth. To spur production, the US federal government has invested in aquaculture programs, grants, and research to support industry growth, including a 2020 presidential executive order encouraging growth of the industry (Malone and Melstrom 2021). In addition, as with most agriculture, there is a need to encourage future growth that is sustainable (Valenti et al. 2018). To effectively promote sustainable growth, it is essential to understand growth barriers in the industry, and therefore which areas to delegate more research, funding, and structural support.

In this study, we work to fill those gaps by addressing two research questions:

1. What level of trust diversity exists in aquaculture production?

2. What does the level of trust in the industry mean for institutional resilience of the industry?

Additionally, we are interested in understanding trust disturbances and repair in this context, so we investigate:

3. What forms of trust are more influential than others?
4. What trust repair strategies could be effective when trust is disturbed?

Methods

To address the research questions, we interviewed aquaculture producers about their trust relationships in the industry. After reviewing prior research, we developed an inductive interview guide (S1 Text) to elicit opinions and comments on trust in aquaculture management and regulation. The guide contained questions about trust and the trustworthiness of information sources, regulators, and Extension. The interview guide was pilot-tested both within the research team and with aquaculture producers from outside of the study region. As a result of pilot testing, we made minor revisions to the interview guide to increase the clarity of the questions.

The research protocol was reviewed and approved by the Purdue University Institutional Review Board, protocol IRB-2020-1842.

Recruitment

Our target population was aquaculture producers in US Great Lakes states. Although there is minimal aquaculture in the actual US Great Lakes, the Great Lakes states are home to the Sea Grant Great Lakes Aquaculture Collaborative, a research-and-outreach-focused organization that provides science-based information to aquaculture producers and consumers. As such, it allowed us to leverage our partnerships to develop relationships with the producers we recruited. We recruited participants through professional networks, including colleagues at the Great Lakes Aquaculture Collaborative. We interviewed 34 producers: five in Illinois, five in Indiana, four in Michigan, four in Minnesota, four in New York, five in Ohio, three in Pennsylvania, and four in Wisconsin. Although reaching saturation was our biggest consideration when determining how many interviews we would ultimately conduct, we were also limited by the number of food fish producers in each Great Lakes state that fit our criteria to be interviewed.

Recruitment took place between March 1 and November 30, 2021. Due to the ongoing COVID-19 pandemic, these interviews took place over Zoom between the months of April and November 2021. Each interview lasted approximately 30–60 minutes. We obtained verbal consent for participation and, with the permission of the interviewee, interview audio was recorded, anonymized, and transcribed.

Given the relatively small size of the food fish industry, the extremely low number of food fish producers in certain Great Lakes states (in some states, we interviewed every known food fish producer), and the need to protect confidentiality, we will not disclose detailed

demographic information about the participants in this study. We will also not disclose the state in which a participant is located when discussing their responses.

Analysis

We transcribed the interviews and loaded them into NVivo version 13 for analysis. As we performed an initial read through each transcript, we drafted a codebook using a mixture of deductive and inductive thematic coding for latent themes grounded in the trust ecology framework. During coding, we greatly expanded the codebook to include semantic themes and subthemes that emerged inductively as we read through each transcript, with some themes fitting nicely into the constructs of the underlying theory and some that did not.

To calculate intercoder reliability, three analysts independently coded the same three interviews using the established codebook, extracting quotes or phrases that corresponded to themes in the codebook. After coding was completed, we calculated Cohen's Kappa using with a coding comparison query in NVivo. The average Cohen's kappa coefficient was 0.81, suggesting substantial agreement between coders (Landis and Koch 1977). Despite the high overall average kappa, there were some individual codes that scored lower than others. Further communication between the coders was necessary to clarify differences in coding, and adjustments to the inclusion criteria for certain themes in the codebook were made to ensure coding consistency and agreement among the coders. With intercoder reliability established and a more nuanced codebook finalized, one analyst (HH) coded all 34 interview transcripts.

Using Stern and Baird's (2015) framework, we coded each of the four trust dimensions in two forms: positive trust and distrust. Positive trust is evidence trust diversity and is a component of institutional resilience. Distrust has both positive (by spurring action) and negative (by reducing support for the management regime) effects and measuring distrust helps us understand which trust dimensions are more susceptible to disturbance in this case, and which should be focused on for repair. We also examine cases of lack of trust, as well as trust repair strategies discovered.

We used the SRQR reporting guideline to draft this manuscript, and the SRQR reporting checklist when editing. The SRQR table is included as supplemental table S2.

Research paradigm and researcher characteristics

Our approach is post-positivist in that this research adapts theory to explain an observed phenomenon. Researcher characteristics can inform all data analysis, including qualitative data analysis. At the time of the study, the researchers were a graduate student and a research faculty member, both of whom were familiar with, but did not actively participate in the aquaculture industry. In addition, the corresponding author (JSC) serves as PI on the Great Lakes Aquaculture Collaborative, which is a National Oceanic and Atmospheric Administration/Sea Grant-funded aquaculture research and outreach project. Our interpretation of the data is inherently informed by these and other researcher characteristics.

Results and discussion

Trust diversity

Trust diversity is achieved through high levels of *trust richness* and *trust evenness*, which can be crucial for institutional resilience (Stern and Baird, 2015). These measures apply solely to trust, not distrust or lack of trust. Trust richness refers to the variety of trust dimensions (dispositional, rational, affinitive, procedural) within a network, while trust evenness denotes the relative abundance of these types.

To measure richness and evenness, we analyzed the frequency of each trust dimension across interviews (Table 1). Among the 34 participants, all four trust dimensions were abundant, indicating high trust richness. The occurrence of each trust type ranged from 26 to 32 interviews. Rational and affinitive trust were the most frequently mentioned, followed by dispositional and procedural trust. This data suggests strong trust evenness, which enhances institutional resilience to various disturbances (Stern and Baird, 2015). Below, we explore key themes related to positive trust and distrust for each trust dimension.

Table 1. Frequency of the dimensions and types of trust in interviews with 34 aquaculture producers.

Trust Type	Number of interviews present
Dispositional	
Trust	29
Distrust	17

Trust Type	Number of interviews present
Rational	
Trust	32
Distrust	17
Affinitive	
Trust	32
Distrust	17
Procedural	
Trust	26
Distrust	14

Dispositional trust

We defined *dispositional trust* using two main codes: *general tendency to trust*, and *general trust in regulators*. The inclusion criteria for both positive dispositional trust and distrust are detailed in S2 Table. The most common theme among interviewees was a cautious approach to information, characterized by taking it with a grain of salt and conducting their own research to validate any information before acting on it. As one producer says,

> "Like anything, you validate whatever you get, you know, I'm going to check with one or two different people and make sure that they're in agreement on it, before

> I use it. So yeah, I do trust what they're giving is the best information to their knowledge. And so, we use it based on that."

We saw this attitude across data extracted for both dispositional trust and distrust, in slightly different ways. For example, some interviewees were generally more trusting whilst still wanting to validate the information, while others tended to not trust the information until they could prove it through their own research. This difference is slight but illustrates how producers on both ends of the spectrum (i.e., those who tend to be trusting versus those who tend not to trust) see doing their own research as an important step in determining the trustworthiness of information.

There do not appear to be high-enough levels of dispositional trust to suggest blind faith or passivity among producers. Instead, there seems to be a healthy level of dispositional distrust that serves a positive function for producers. They are actively seeking to ensure the validity of information before putting their money on it, potentially due to the high-risk nature of aquaculture, as one interviewee expresses:

> "I don't ever trust anybody's information 100%. I don't put my bread and butter on somebody else's word. So, you take their concept, and you try it. And if it proves out true, you flush out-- everything in aquaculture, you try it. You don't use your bread money to try it with, you got to try it with money that you can afford to lose. And you can assume that it's gone. And then you try a concept, or you try a system or strategy, and you just write that off as gone as you begin to try it. If it works great then you've got something, and if it don't...you can't use your bread money to try anybody's information. Nothing's going to be what the textbooks tell

you. Just nothing is...don't do it with your bread money, don't put your house mortgage on the line, because you'll lose it. There's a lot of unknowns, you know."

This attitude of not trusting anyone 100% was common within the dispositional distrust data, which many interviewees associated with negative past experiences related to trusting the information of others regarding aquaculture.

Rational trust

For *rational trust*, we used two main codes: *trust based on the perceived cost or benefit of trusting*, and *trust in reputation or perceived competence*. The inclusion criteria for each code for both positive rational trust and distrust can be found in S3 Table.

Producers often expressed rational trust or distrust based on the trustee's relevant experience (or lack thereof). For example, many producers expressed only trusting information from trustees who are not only in the aquaculture business but ideally are specifically raising the same species in the same system as the trustor. One interviewee cautioned that *"...just because it works for them, their water, their pond, or their indoor system or their situation, that doesn't just guarantee that it's gonna work for you. That's the distrust portion."* Being in aquaculture or producing the same fish were not sufficient to justify trusting, though: they typically needed to be combined with a positive reputation and high levels of perceived competence of the potential trustee. As one producer explained when asked who they trust for information,

"Yes, there's a couple of other fish farmers. One in particular in the state that has been in aquaculture for quite a few years, has a formal education in aquaculture,

> has worked for other companies, and then he's operated his own farm for several years. And he's just an excellent resource for me, I find myself talking to him quite a bit."

This was not an issue of shared identity and affinitive trust, but rather a calculated cost/benefit analysis that trusting information from others who have a completely different operation could result in losses or failure.

This distrust also extends to academics and/or regulators who some producers feel do not have the "hands-on" experience with being a fish farmer that is required to be deemed a trustworthy source of information regarding raising fish. When asked if they trust Extension personnel, one interviewee responded,

> "Yes, I would, to some extent, but mostly...you now, there's a big difference between a college education and a hands-on experience. And the biggest thing that I've seen with raising fish is watching, observing. If you see any different behavior, like for instance, flashing, they're getting parasites. So, we need to treat that you know, people can look at books and whatever, but till you have a hands-on experience, and have done this, you probably are not going to be able to help me."

Despite the presence of distrust, most interviewees expressed that universities and academic products were trustworthy sources of information in general. Many expressed trust in the reputation and credibility of academic research and knowledge, a concept that we explore under procedural trust below. It seemed to be more of a case-by-case basis as to which types of information can be trusted and relevant coming from academic sources,

and which cannot. It should also be noted that some interviewees who expressed distrust in academic services such as Extension programs also admitted to not having any experience working with them.

Another key theme we identified was a widespread distrust in people who are trying to 'sell them something.' One interviewee expressed that *"as far as the trust factor, somebody who is selling something is probably the least likely for me to align with and trust."* This was a widely held notion among the interviewees, with another claiming that *"If you were just trying to sell something, I immediately disregard them as untrustworthy."* This distrust seems to be a result of a perceived misalignment of goals between the trustor and the trustee, where producers feel like their best interests and success are not at the heart of the salesperson, and that their end goal is just to make money.

Affinitive trust

For affinitive trust, we used three main codes: trust based on positive shared experiences, trust based on feelings of social connectedness or shared values and identity, and trust based on perceptions of the character or integrity of the trustee. The inclusion criteria for each code for both positive affinitive trust and distrust can be found in S4 Table. Affinitive trust seemed to have a large influence on the interviewees and their perceptions of trustworthiness. Our interview data illustrated how impactful affinitive trust can be on relationships in aquaculture, for better or worse.

The most prevalent theme we identified regarding affinitive trust was the emphasis on shared values as a key determinant of trust. This theme was evident in respondents'

Atlantic salmon

Sets the standard for aquaculture breeding.

- Virtually *all* Atlantic salmon in production is genetically improved
- An *exceptional* situation within aquaculture

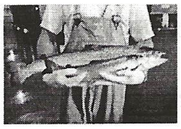

Carps

Am of interest to me. My great-grandfather (maternal) was a karp farmer in Poland and the family name was Karp!

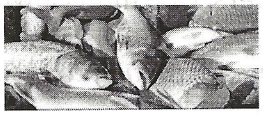

- Most-raised species complex world-wide — shown in orange
- Have been raised in China for 2,000 to 4,000 years

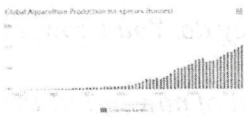

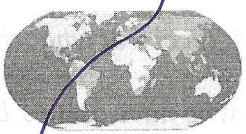

Though the sector has grown rapidly on part to [illegible] with the rise of aquaculture science

Carps

- Two major Asian *polyculture* systems: — i.e. multiple species raised in one pond to take advantage of all aquatic products
- Chinese major carps: include a
  - Silver carp - phytoplanktivore
  - Bighead carp - zooplanktivore
  - Common carp – benthic invertivore
  - Grass carp - herbivore
- Indian major carps:
  - Catla – surface foods
  - Rohu - herbivore
  - Mrigal – detritivore
- Common carp raised in *monoculture* in Europe
- Programmatic genetic improvement for common carp only so...
- *Let's focus on common carp*

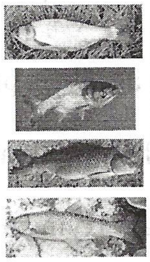

> agencies, and have been since the day we got into this business. You know, anytime you try to do something new, something different, something better, Somebody's got a problem with it."

Negative past experiences or feelings of social disconnectedness often led to this distrust, along with feelings that their regulators do not want to see them succeed as a business or an industry.

Feelings of social connectedness and shared identity was a prevalent theme, particularly in relationships between aquaculture producers. Potentially due to the relatively small size of the industry and the challenges that come with being an aquaculture producer, many respondents felt connected to other industry members and formed stronger trust relationships. As one interviewee put it,

> "I've got some serious role models in this industry. And, you know, people that have been farming for 50–60 years, these people have gone through all the trials and tribulations that I've gone through, and instead of me making the same mistake, I can ask them, and they'll tell me, based on their experience. What I think is new to me, they've already done it five times. And what's nice about our industry, we're small enough, we all kind of know each other, and we become lifelong friends and colleagues. We're not competitors. So, I have some amazing-- and some of these legends of the industry, they'll take my phone call right now. They'll stop what they're doing, because they're so pro-industry, that if I have a problem at my little farm, these people will take my call...I trust people who are in the industry."

We also saw a theme carry over from rational trust where we saw distrust based on a misalignment of goals between the trustor and potential trustee. Similarly, in the context of affinitive trust, we saw a strong emphasis on shared values (or lack thereof) as a determinant of trust. Many interviewees mentioned an affinitive distrust in others who they perceive to be *"super greedy," "money-hungry,"* or *"venture capitalists"* who are *"going after the almighty dollar"* by pushing faulty systems or making false promises to other industry members that end up failing to prove successful in the long run. As one interviewee put it, *"There's some very good experts out there. But there are more quote unquote experts that are expert at taking your money and mediocre at putting systems together that work every single day."* Many producers described with these types of people, leading them to be cautious and untrusting, for good reason. This distrust is likely serving a positive function by protecting producers from failure, but nonetheless poses risks for some producers who do not have these past experiences to draw upon when encountered with a similar situation. It also raises questions about how these types of people affect the growth of the industry, and how pushing unreliable systems could further damage the reputation of aquaculture and contribute to the already low levels of trust held by banks and lenders.

Shared values (or the lack thereof) emerged as a crucial determinant of trust. Many interviewees expressed affinitive distrust towards individuals perceived as *"super greedy," "money-hungry,"* or *"venture capitalists"* who prioritize profit over integrity by promoting faulty systems or making false promises that ultimately fail. As one interviewee noted, *"There's some very good experts out there. But there are more quote unquote experts that are*

expert at taking your money and mediocre at putting systems together that work every single day."

Procedural trust

For *procedural trust,* we used three main codes: *trust in systems, trust in academia and research,* and *trust in the regulatory process or entity*. The inclusion criteria for both positive procedural trust and distrust are detailed in S5 Table. Our interview data revealed various strengths and disturbances in procedural trust, as well as opportunities for top-down repair. Procedural trust and distrust were directed at different structures and systems within the aquaculture industry, primarily including regulatory agencies and processes, aquaculture associations, and academic/research entities.

One of the main procedural trust themes was the regulatory process. Higher levels of procedural trust in the regulatory process were typically due to perceived consistency and adherence to the "rules" by the agencies. Conversely, distrust arose from perceived inconsistencies and rule violations. Notably, there was a discrepancy between the procedural trust interviewees had in federal regulatory agencies versus state or local agencies. One producer mentioned trusting the FDA more than their state's DNR because,

> "I can read the rules, and they can read the rules, and we interpret the rules the same. So, it's a little bit more black-and-white. The Department of Natural Resources, they kind of interpret the rules differently. And they operate in that little bit of a gray area…so that's the difference. I mean, it's point blank, the FDA, they come in, they're nonbiased. DNR is biased."

We also saw high levels of distrust regarding the regulatory process due to perceived inconsistency of the process, high levels of turnover within the regulatory agencies, and a lack of engagement with producers throughout the process. Inconsistency was mentioned constantly in our interviews, with some producers claiming that "*the bigger challenge is really the regulatory environment. You never know how that's gonna change and how it might make it hard for you to be profitable and do business. So that's my biggest concern is, is the regulatory environment.*" This notion was expressed by others as well, some suggesting that:

> "Their rules and regulations change all the time. One year I apply for a permit, no problem. Next time I go to apply for a permit? 'Well, you got to add this to it.' Well, how long was I supposed to be doing that? 'Well, you were supposed to be doing it the whole time.' So why didn't anybody catch it for the last three years, but you caught it on year four? There's no penalty, but they're just like, you know, it's kind of like calling the Extension agency and you know, every time you call, you get four different people. Well, same thing. You call government, you know, entity, it's not the same person every time. Just, what I want is, I want the same piece of paper in front of whoever walked up to that desk for that day to pick up the job is that they have the same sheet of paper that the next guy had, and everything is exactly the same. And I just, I don't feel like that happens."

Procedural inconsistency could come from turnover within agencies or a lack of communication between regulators, making the process more confusing and difficult for

the producer to navigate. Although turnover is sometimes inevitable, creating streamlined or more straightforward processes for regulation could strengthen procedural trust.

Groups such as aquaculture associations, councils, and boards were brought up frequently in our interviews as entities and avenues through which producers can connect with each other and with relevant entities such as their regulatory agencies. When these groups are functioning properly, many respondents felt positively trusting of them and claimed to lean on them routinely for information about aquaculture. However, there were several interviews where participants expressed concern that such structures were not functioning properly. In the example below, one interviewee shares their experience of regulators pushing through new regulations without input of producers, despite having a council that was designed for this reason:

> "So, it's just, that's what it was designed for. To have an avenue where we can have maybe some direct contact with Department of Ag, direct contact with DEP, direct contact with the Fish Commission. That's why this council was put together. And there's a lot of farmers that sit on it. Through different industries, like you know, an ornamental fish farmer, that sits on it. There's fish producers, fish processors. There's vendors in the industry, like feed, feed reps and things like that. But that's what it was designed for. But it just doesn't seem like it's working the way it should."

Other participants shared similar experiences and expressed interest in strengthening these groups so that they serve the function that they were designed to serve, which would

foster stronger procedural trust in the industry and potentially help develop other types of trust as well.

Many interviewees spoke about their trust in academic research or Extension, expressing trust in the process of generating knowledge through academic research. Some respondents mentioned relying on or anticipating future academic research to learn about the most up-to-date advancements in the industry, usually regarding production. One interviewee described their trust below:

> "So that's why I rely heavily on a university type program, where universities have intellectual property they license, they're typically independent of manufacturers and you know, that to me is a nice source of information. They're not biased. They're not production-minded, as a rule."

Procedural trust in academia as a system also precedes a form of rational trust that many producers expressed regarding people in the industry who have strong academic backgrounds in aquaculture. Many felt academic backgrounds were representative of competency and expertise, as one interviewee said *"really, who I rely on, and I kind of have a network of people working with, is mostly experts in the field, and they generally come from university settings"*.

However, we also found several forms of procedural distrust of academia. Some responses expressed concern that academic study of aquaculture does not translate to "real-life" operation of an aquaculture farm. Other responses pointed to deeper concerns with academia, such as worries that knowledge generated by academic research is not shared with producers for the benefit of the industry. One producer explained the situation,

> "…they just got funded to do a study, which is really good. We need that information. But more importantly, we need that information to be shared. And that's where the breakdown comes into play with the higher education research facilities, is that they're only interested in patents…they have had no public disclosure, after taking millions of dollars in federal and state funding grants and have done zero to move any valuable information into the public sector. And I want that to be extremely clear to you people because you're the ones that are that are gathering this information, and that information needs to be brought to light. Because these people are taking serious federal dollars and are not doing anything with it. They're just funding their own operations."

This is an example of how procedural distrust could threaten to lower other forms of trust as well. Making useful information accessible to producers could address this type of distrust and help to rebuild trust and move the industry forward.

Lack of trust

Lack of trust was observed among producers who had limited interaction with potential trustees, preventing them from developing a sense of trust. For instance, states without a strong aquaculture Extension presence were often associated with producers expressing a lack of trust in Extension. Producers clarified that this was not distrust, but rather an absence of trust, suggesting that a larger presence would likely encourage collaboration. Similarly, when asked about their most important regulators and their trust in them, one farmer noted: *"We haven't had much interaction with them. We just do our transport licensing through them. That type of thing."*

In our data, lack of trust appeared to serve a neutral function. The instances of lack of trust we identified ~~were most~~ly workable baselines for building trust, with many respondents showing openness to potential trustees. This seems connected to the relatively high levels of dispositional trust found in our data. With more information and opportunities to engage, lack of trust could potentially be transformed into stronger trust relationships.

Trust scale

The scale of trust—individual, group, organization, etc.—is another important concept in the trust ecology framework (Stern and Baird 2015). Trust, distrust, and lack of trust can exist at various scales in a network, and trust may operate differently at different scales (Carlton et al. in review). This theme came up frequently in our interviews.

One example is that of producers' trust in regulators. Some producers expressed a procedural distrust in the regulatory process or entity as a whole but still exhibited positive affinitive trust in the individual regulators that they've worked with in the past within that greater entity. One interviewee explains this difference below:

> " Well, you know, DEP, they're a regulator, so, it's always a concern, what happens there, what the motives are...you're on the different side. Fish and Wildlife Service, they're a resource agency, they have a different charge and a different way of looking at things. So, I don't always trust because I'm not sure where they stand. But even having said that, there's people within those agencies that I trust completely. Just depends on the situation, the issue, because, you know, you're gonna come from different angles. And that's how it is."

This example illustrates how trust scales differ and how one dimension of trust can buffer against low levels of another: here, affinitive trust toward an individual might mitigate lower levels of larger-scale procedural trust.

Trust relationships between producers and Extension also vary by scale. For instance, producers in states with a small aquaculture Extension presence often exhibit a general lack of trust in Extension agents due to limited interaction. However, these producers may still hold positive procedural trust in the Extension process, viewing it as a useful and trustworthy source of information if more accessible. Thus, the general lack of trust can serve as a foundation for building stronger rational and affinitive trust through increased Extension presence and personal relationships.

Similarly, many producers expressed low affinitive trust in academic researchers due to their inaccessibility. One respondent noted, *"A lot of researchers, they're just great researchers, but they don't want to be bothered or don't want to deal with people. And even though their livelihood depends on us, because that's how they justify funding programs that they use..."*

However, the same respondent expressed procedural trust in academic research and preferred accessing this information through Extension personnel, whose role is to be approachable and connect academic information to producers.

Trust repair

Our interviews suggest two general methods for **trust repair** in instances where there is distrust: **distrust regulation** and **trustworthiness demonstration** (Table 2).

Table 2. Trust repair methods identified in interviews with 34 aquaculture producers.

Trust Repair Method	Function	Examples from interviews
Distrust regulation	Prevents future loss of trust by addressing the causes of previous trust breakage	Involve producers in creation of regulations; Educate regulators about aquaculture; Streamline regulatory process; Make research accessible to producers; Establish supportive structures for aquaculture producers; Include producer needs in research goals; Reduce turnover in agencies
Trustworthiness demonstration	Incorporates affinitive and rational elements to display trustworthiness	Apologize and take responsibility for wrongdoing, offer reparations; Advocate for aquaculture industry success; Demonstrate aquaculture knowledge and expertise; Communicate shared values and goals; Build trustworthy face-to-face interpersonal relationships; Strengthen extension presence

Distrust regulation, which prevents future loss of trust by addressing the issues that caused a breakage, emerged in several forms during our interviews. Participants suggested that

trust could be increased by involving producers in altering or creating regulations, educating regulators about aquaculture, streamlining the regulatory process to reduce confusion, and minimizing turnover in regulatory agencies.

Regarding producer support by regulators or resource agencies, many interviewees expressed a desire for structures to help aquaculture farms succeed and mitigate high risk levels. Examples include federal grant money or insurance programs similar to those for other forms of agriculture. Additionally, many participants highlighted unmet research needs that universities could better address and expressed a desire for greater access to aquaculture research knowledge.

Trustworthiness demonstration, involving affinitive and rational trust repair, also emerged. Many producers emphasized the importance of affinitive trust, valuing it highly. They believe that individuals and entities that have demonstrated a lack of trustworthiness should take responsibility, apologize, and potentially offer reparations. Building strong, face-to-face interpersonal connections is crucial, especially where trust is lacking. This includes practicing stronger communication and explicitly expressing shared goals and values so that producers feel supported.

Additionally, producers want federal and state agencies to be stronger advocates for aquaculture, demonstrating shared values in the success of their businesses and the industry. It is also important to producers that regulators and other entities demonstrate competence and possess higher levels of aquaculture knowledge.

Summary and conclusions

By measuring the richness and evenness of trust in our interview data, we observed high levels of trust diversity, suggesting that aquaculture as an institution is resilient to many disturbances. Each trust type had unique challenges and strengths, often shared among respondents.

Overall, the data suggest that affinitive trust is the most influential in the trust relationships we examined. Rational trust also played a significant role, as producers relied heavily on the experiences and background of potential trustees to determine their credibility. Dispositional trust, particularly distrust, served a positive function by motivating producers to "do their homework" before trusting. Procedural trust, especially between producers and regulatory processes, varied and offered opportunities for trust repair. Additionally, trust sometimes varied by scale or trustee, such as with producers who had procedural distrust in a regulatory agency as a whole but strong affinitive and rational trust with an individual regulator.

Dispositional trust

Dispositional trust, or the baseline from which trust can be built or eroded, appeared workable in our interview data. The most prevalent theme was to "do your own research." Many participants tended not to trust until they could validate or verify the information provided. This does not necessarily indicate high levels of distrust, but rather a lack of trust that can be built upon with sufficient information. However, dispositional distrust was still

common, with many interviewees associating it with negative past experiences related to trusting others' information about aquaculture.

Despite some producers' tendency to distrust, our data suggests an open-mindedness and willingness to trust if provided with necessary proof, such as demonstrated success or verified sources. Additionally, the tendency to distrust could be mitigated by strengthening the other three trust types. The data suggested healthy levels of distrust that motivated producers to be active and engaged in their relationships rather than passively trusting.

Rational trust

Rational trust was highest in our data when individuals perceived a positive reputation along with high levels of relevant experience and competence in a potential trustee. This aligns with the trust ecology framework of Stern and Coleman (2015) and Stern and Baird (2015). Interviewees felt most trusting of information from sources with experience and success in aquaculture, particularly those growing the same species or using similar systems. Many emphasized the uniqueness of each aquaculture operation, noting that regardless of an individual's reputation, their information and practices might not be applicable to another farm. Similarly, many distrusted academics without "real" experience running a fish farm, feeling that university knowledge differs from lessons learned on a farm where livelihoods are at stake.

The most common disturbance for rational trust was a misalignment of goals between the trustor and trustee, or a perceived lack of relevant experience or competence. Specifically, many distrusted vendors or anyone trying to "sell them something," viewing them as the

~~least~~ trustworthy. This was interpreted as a misalignment of goals and potentially a negative reputation of the trustee. Low levels of rational trust could be strengthened by clear communication of goals and demonstrations of competence and success. According to the trust ecology framework, these disturbances could also be buffered if other trust types are high, reducing the need for rational trust.

Affinitive trust

Affinitive trust had the strongest influence on perceptions of trustworthiness among interviewees. Our data suggested that shared values, particularly regarding the success and growth of aquaculture, were crucial determinants of affinitive trust across several relationships.

Producers expressed affinitive distrust when they felt the trustee was not an advocate or supporter of the industry or was actively against its growth. This distrust was often linked to negative past experiences or feelings of social disconnectedness, leading producers to believe that their regulators or other potential trustees did not respect or value their success. This issue has significant potential for damage control through trust-repair strategies.

Feelings of social connectedness and shared identity were also highly important in trust relationships between aquaculture producers. This theme highlights the pride many producers have in their identity as fish farmers, which can lead them to trust other producers based on mutual respect and understanding of each other's experiences. Many

respondents noted low levels of competition in the industry, with generally positive and supportive relationships among producers.

This data suggests that aquaculture producers place high importance on affinitive trust. Groups interacting with the aquaculture industry should consider this, as building strong interpersonal relationships is imperative to gaining producers' trust. Low levels of affinitive trust could threaten trust diversity and institutional resilience. Situations where affinitive trust is negatively impacted should be prioritized for trust repair. If trust-repair strategies are not employed promptly and effectively, it could further damage the perceived integrity and social connectedness of the trustee.

Procedural trust

Procedural trust in our interviews was most discussed in the context of the regulatory process. It seemed to result from feelings that the "rules" were clear, consistent, and adhered to by regulators. Federal regulations often received more procedural trust than state or local regulations due to the perception that federal processes were more "by the book" and consistent, regardless of factors like employee turnover.

Procedural distrust often stemmed from perceived inconsistencies in the regulatory process, such as changing regulations or employee turnover causing confusion for both producers and regulators. Many interviewees desired a more streamlined and standardized regulatory process, believing it could alleviate some of the growing pains associated with employee turnover in regulatory agencies. A clear, consistent, straightforward, and

unchanging process would help ensure that producers and regulators understand what needs to be done, regardless of who holds a given position.

Also, some interviewees attributed their procedural distrust in regulatory agencies to past experiences where producers were not included or engaged in changing or introducing regulations. This was particularly problematic given the existence of councils and boards designed for engagement and communication between producers and regulators. These groups play a crucial role in reinforcing procedural trust. If they are dysfunctional, as some producers felt, they should be a focus for trust repair strategies to strengthen both procedural and affinitive trust.

Trust repair

Trust repair efforts are crucial for overcoming negative expectations and restoring or creating positive expectations about future trust relationships (Stern and Baird 2015). We examined two types of trust repair: distrust regulation and trustworthiness demonstration (Gillespie and Dietz 2009). These strategies, suggested by producers regarding issues in their own trust relationships, are important to consider. This data offers unique insights into their personal trust dynamics and how they believe trust can be rebuilt with regulators, academics, Extension, other producers, and beyond. To be most effective, these strategies should be the primary focus of trust repair efforts.

Limitations

This study had several limitations. As a qualitative study, the findings are not intended to be generalizable to the broader population of aquaculture producers. Indeed, our

purposive sampling method, while appropriate for this study, prevents such generalization. In addition, our analysis relies on the interviewee's answers, which are self-reported data from semi-structured interviews. These are subject to recall, social desirability, and other biases.

Conclusions

Our analysis of trust dynamics in aquaculture using the trust ecology framework provided valuable insights into trust relationships. We found a high level of trust diversity, indicating strong institutional resilience. We also identified the most prevalent types of trust among aquaculture producers, what they value in trust relationships, and how they believe trust can be effectively repaired. Data on lack of trust and distrust further deepened our understanding of trust dynamics in aquaculture.

As with many studies on trust dynamics in natural resource management, our data emphasizes the importance of building stronger interpersonal relationships between regulatory agencies and producers. Additionally, our research shows that producers feel a need for more vocal advocacy for the industry within the regulatory system, stressing the importance of growth as demand for seafood rises with the population. By developing trust and developing relationships, the aquaculture industry may come closer to achieving its potential.

Should this methodological approach be applied in other aquaculture/regulation contexts? Is that why this one should be published in an international journal?

Acknowledgements

The authors ~~want to~~ acknowledge Drs. Zhao Ma, Trey Malone, and Kwamena Quagrainie, the members of the Great Lakes Aquaculture Collaborative, and B. Chavis for their support on this project.

Conflicts of interest

The authors acknowledge no conflicts of interest in this study.

Availability of data and material

Redacted interview transcripts are available at the Purdue University Research Repository. The citation is: Carlton, J. S.; Hartenstine, H. (2026). Qualitative interviews of Midwest aquaculture producers. Purdue University Research Repository. doi:10.4231/73HR-5K71.

Ethics

This study was reviewed and approved by the Purdue University Institutional Review Board, protocol IRB-2020-1842.
