# Supplementary material for: Trust dynamics among aquaculture producers and regulators in the midwestern United States
Source: PLoS One. 2026 Oct 15;21(10):e0358442. doi: 10.1371/journal.pone.0358442 (PMC13671553; doi:10.1371/journal.pone.0358442)
Supplement: Supplementary file 3 [file pone.0358442.s001.docx]

**S1 Text. Interview guide.**

Below are the questions related to trust, excerpted from a larger interview guide that also touched on several topics not discussed in this manuscript. For the entire interview guide, please see Appendix A in Hartenstine, Haley A. Land-based aquaculture in the Great Lakes region: Trust dynamics and barriers to growth. M.S. Thesis, Purdue University, West Lafayette, Indiana, 2022.

a. If you wanted more information specifically about expanding your aquaculture business, who would you turn to first? Why?
   i. Do you have past experience with ____________?
b. In the past, who have you relied on most for information relating to aquaculture?
   i. What makes them trustworthy?
c. Are there people who you **don’t** trust for information related to aquaculture? Why?
d. Do you trust Extension for information about aquaculture? Why or why not?
   i. What could they do to increase or decrease your trust?
   ii. **If they don’t talk about their past experience with Extension, ask:* Do you have past experience with ____________?
e. Who is the most important regulator of your aquaculture business?
   i. Do you trust them? Why or why not?
   ii. What could they do to increase or decrease your trust?
f. Are there other important regulators of your aquaculture business? Do you trust them? Why or why not? *Keep asking until all important regulators are discussed*.
g. Do you think aquaculture is more or less regulated than other farmed protein sources?
