# Supplementary material for: Trust dynamics among aquaculture producers and regulators in the midwestern United States
Source: PLoS One. 2026 Oct 15;21(10):e0358442. doi: 10.1371/journal.pone.0358442 (PMC13671553; doi:10.1371/journal.pone.0358442)
Supplement: Supplementary file 5 [file pone.0358442.s003.docx]

**S2 Table. Dispositional trust types, inclusion criteria, and relevant data from the interviews.**

| Trust types | # of interviews | Inclusion criteria | Exemplary quotes |
| --- | --- | --- | --- |
| **Trust** | 29 |  |  |
| General tendency to trust |  | Mention of general tendency to trust in others, context independent. May trust until given a reason not to. | “Um, yeah…I feel they are trustworthy. I just don’t know how to…I guess you just assume they’re trustworthy.” |
| General trust in regulators |  | Mentions overall tendency to trust in regulators and other oversight entities. Context independent. | “Yes (I trust them). They give me no reason not to.” |
| **Distrust** | 17 |  |  |
| General tendency to distrust |  | Mentions general tendency to distrust in others, context independent. May “not trust anyone” or only themselves. “Do your own research” attitude. | “Bottom line is, I don’t trust anybody for information unless they’ve demonstrated in some way that I can actually trus them. I’ve been around long enough, and I guess I’m savvy enough to smell a skunk when I, you know, when they’re around. So, I proceed with extreme caution. And we’re just very careful about what information we actually believe or what we don’t.” |
