# Supplementary material for: Trust dynamics among aquaculture producers and regulators in the midwestern United States
Source: PLoS One. 2026 Oct 15;21(10):e0358442. doi: 10.1371/journal.pone.0358442 (PMC13671553; doi:10.1371/journal.pone.0358442)
Supplement: Supplementary file 6 [file pone.0358442.s004.docx]

**S3 Table. Rational trust types, inclusion criteria, and relevant data from the interviews.**

| Trust types | # of interviews | Inclusion criteria | Exemplary quotes |
| --- | --- | --- | --- |
| **Trust** | 32 |  |  |
| Trust based on cost/benefit of trusting |  | Mentions calculated or perceived benefit of trusting. Predicted outcome of trusting is beneficial, leading to trust; Alignment of goals; Trustee’s experience doing relevant work (e.g. farming same species or same system type) | “They’re actually hands on, have a laboratory or have a fish farm or have some direct connection to working with fish…And so I’ve gotten my best advice from people who actually do hands on work.” |
| Trust in reputation/perceived competence |  | Mentions reputation of the trustee, as a successful business, reputable educational background, and/or their general perceived competence. | “…one in particular in the state that has been in aquaculture for quite a few years, has a formal education in aquaculture, has worked for other companies, and then operated his own farm for several years. And he’s just an excellent resource for me, I find myself talking to him quite a bit.” |
| **Distrust** | 17 |  |  |
| Distrust based on cost/benefit of trusting |  | Mentions calculated or perceived cost of trusting. Predicted outcome is costly or harmful of trustor, leading to distrust; Misalignment of goals (e.g. someone selling something). Trustee’s lack of experience doing relevant work (e.g. farming different species; academics/lab work) | “And there’s a lot of bad information out there. The reason why the information on the internet is not true is everybody’s got their own water source. What works here–my information, my data– will not work down the road.” |
| Distrust in reputation/perceived competence |  | Mentions reputation of the trustee; negative reputation as a business, a lack of education, and/or general perceived incompetence. | “There are certain people who think they know what they’re talking about who have not got the experience necessary to really be the expert that they think they are and they have led us down in the blind alleys a couple of times. They’ve done it because they didn’t know, but that makes you not trust their judgment or their words.” |
