# Supplementary material for: Trust dynamics among aquaculture producers and regulators in the midwestern United States
Source: PLoS One. 2026 Oct 15;21(10):e0358442. doi: 10.1371/journal.pone.0358442 (PMC13671553; doi:10.1371/journal.pone.0358442)
Supplement: Supplementary file 7 [file pone.0358442.s005.docx]

**S4 Table. Affinitive trust types, inclusion criteria, and relevant data from the interviews.**

| Trust Types | # of Interviews | Inclusion Criteria | Exemplary Quotes |
| --- | --- | --- | --- |
| Trust | 32 |  |  |
| Trust based on positive shared experiences |  | Mentions trust based on positive shared experiences with the trustee | “And we’ve had the USDA here, a couple of times, I’ve gotten some great, I’ve got a great relationship with the local agent, she’s been very helpful in, in passing along information that she has seen in other operations to help me in my operation.” |
| Trust based on social connectedness, shared values, or identity |  | Mentions trust based on feelings of personal/social connection to the trustee; Trust based on shared values or shared identity (e.g., fish farmer, political views) | “Open mindedness, motivation, same values, principles, goals that I have. I want to help people out and, we’ve had people that have stopped here…I got to think how to say this…I want to align myself with people that have got the same principles and goals as what I do.” |
| Trust based on character or integrity |  | Mentions a positive perception of trustee’s character or integrity, resulting in trust. | “…the farmers that I mentioned, all have a real high level of integrity, they’re really good people. So I first look up to that, their ethics is untouchable. And then their business success. I look up to the thing that they’ve done to be successful.” |
| Distrust | 17 |  |  |
| Distrust based on negative shared experiences |  | Mentions distrust based on negative shared experiences with trustee. | “But with regard to our, you know, when we first got our farm started, they came in very strong, rather than trying to help us navigate, how to make sure that we were doing everything properly. They just kind of showed their muscles and said, you know, if you don’t do this, we’ll shut you down.” |
| Distrust based on lack of social connectedness, shared values, or identity |  | Mentions distrust based on a feeling of personal/social disconnection to trustee; Distrust based on lack of shared values or lack of shared identity. | “No [I don’t trust them], because they’re all republicans. There’s no democrats except me. No, I don’t look up to any of them, they all want to get big, they all want to be millionaires. You know, come on be satisfied with a nice income and a beautiful place to live. We love where we live.” |
| Distrust based on lack of character or integrity |  | Mentions a negative perception of the trustee’s character or integrity, leading them not to be trusted. | “I just think that there are some very compelling sales people out there that are focused more on their personal wealth then on how they accomplish that. Integrity, you know, that’s a fundamental. They may think they’re good at it, and they’re just bad…go talk to their customers and find out the facts.” |
