# Supplementary material for: Trust dynamics among aquaculture producers and regulators in the midwestern United States
Source: PLoS One. 2026 Oct 15;21(10):e0358442. doi: 10.1371/journal.pone.0358442 (PMC13671553; doi:10.1371/journal.pone.0358442)
Supplement: Supplementary file 8 [file pone.0358442.s006.docx]

**S5 Table. Procedural trust types, inclusion criteria, and relevant data from the interviews.**

| Trust Types | # of Interviews | Inclusion Criteria | Exemplary Quotes |
| --- | --- | --- | --- |
| Positive Trust | 30 |  |  |
| Trust in systems |  | Interviewee mentions systems through which an avenue is created between them and other entities or individuals that creates or reinforces trust, and they trust that these systems are functional. | “Yeah, they always have been, I mean, I’ve always sat on a lot of boards. So there’s always knowledgeable people. And it depends, in fact, that’s been the the single biggest benefit for being on those boards has been that group of people I’ve gotten to know and that I can call on.” |
| Trust in academia or research |  | Interviewee mentions trusting in the academic/scientific process or research in general. | “Universities, I think, have the patience, you know, that, that maybe some producers might not have. And so I see them as just great resources. And then they can throw a whole lot of talent at observation, and documentation, which I think is really important for our industry, too.” |
| Trust in regulatory process or entity |  | Farmer mentions a trust in the regulatory process as a whole, or regulatory entities. May refer to fuctionality, consistency, fairness, etc. | “[I trust the USDA more than the DNR because] their positions, you know, are usually etched in stone, even if it’s from different administration changes…usually, because that’s kind of regulated for all food industries. So they’re more, you know, they don’t shift around as much, and more of it is based on scientifics.” |
| Positive Distrust | 14 |  |  |
| Distrust in systems |  | Interviewee mentions a distrust of systems through which an avenue is supposed to be created between them and other entities or individuals that creates or reinforces trust, and they do not trust that these systems are functional. | “That’s what it was designed for. To have an avenue where we can have maybe some direct contact with Department of Ag, direct contact with DEP, direct contact with the Fish Commission. That’s why this council was put together. And there’s a lot of farmers that sit on it, through different industries…but it just doesn’t seem like it’s working the way it should.” |
| Distrust in academia or research |  | Interviewee mentions distrust in the academic/scientific process or research in general. | “No offense, but some of these researchers and all this science bullshit. These people are, are in science fair labs, they’re doing cute little projects. They’ve never spent their own money. They don’t know how to do it. They would just do research to get grant money and it’s all, I totally think it’s bullshit. So, you know, they can come and tell me something, but until they put their money where their mouth is, I have no interest.” |
| Distrust in regulatory process or entity |  | Farmer mentions a distrust in the regulatory process as a whole, or regulatory entities. May refer to dysfunction, inconsistency, unfairness, etc. | “I don’t trust them. I mean, there are individuals within the agency that I trust to tell me the truth. There are individuals within the agency that I believe are straight shooters, but the agency as a whole, culturally, and missionally, I don’t trust.” |
