# Supplementary material for: Trust dynamics among aquaculture producers and regulators in the midwestern United States
Source: PLoS One. 2026 Oct 15;21(10):e0358442. doi: 10.1371/journal.pone.0358442 (PMC13671553; doi:10.1371/journal.pone.0358442)
Supplement: Supplementary file 9 [file pone.0358442.s008.docx]

**Dear Editor,**

Thank you for the opportunity to review this manuscript. Please find below my constructive comments for improvement.

**Manuscript Title:** Trust dynamics among aquaculture producers and regulators in the Midwestern United States

**Manuscript Number:** PONE-D-26-14856

**General Comments**

This is a well-written and valuable manuscript that applies the trust ecology framework to an important and understudied context. The qualitative approach yields rich insights, and the findings are clearly presented and connected to the framework.

**Abstract**

**Comment 1**: Briefly note the novelty of applying the trust ecology framework to aquaculture to strengthen the abstract's opening.

**Comment 2**: Briefly hint at *why* stronger relationships and advocacy matter (e.g., for policy experimentation or sustainable growth) to increase impact.

**Introduction**

**Comment 3**: Add a bridging sentence explicitly connecting the challenges of farmer-expert relationships to the trust ecology framework.

**Comment 4**: Briefly note that the regulatory environment is a known barrier to U.S. aquaculture growth to better set up the research questions.

**Comment 5**: Integrate the four research questions more smoothly into the final narrative paragraph of the introduction.

**Methods**

**Comment 6**: Briefly explain how you distinguished "positive trust" from "distrust" during coding.

**Results and Discussion**

**Comment 7**: Briefly note potential downsides of strong in-group trust in the affinitive trust section (e.g., "us vs. them" perceptions).

**Comment 8**: Consider briefly linking regulatory inconsistency to effects on innovation or new entrants.

**Comment 9**: Briefly link each repair strategy in the trust repair section to the trust dimension it targets.

**Summary and Conclusions**

**Comment 10**: Add slightly more specificity about what "advocacy" might look like in practice (e.g., liaisons, lobbying).
