# Supplementary material for: Trust dynamics among aquaculture producers and regulators in the midwestern United States
Source: PLoS One. 2026 Oct 15;21(10):e0358442. doi: 10.1371/journal.pone.0358442 (PMC13671553; doi:10.1371/journal.pone.0358442)
Supplement: Supplementary file 10 [file pone.0358442.s011.docx]

# Response to Reviewers

**Manuscript:** PONE-D-26-14856 — *Trust dynamics among aquaculture producers and regulators in the midwestern United States*

**Authors:** Haley A. Hartenstine and J. Stuart Carlton

We thank the editor and both reviewers for their careful and constructive reading of our manuscript. The comments were detailed and specific, and addressing them has improved the paper considerably. Below we respond to each comment in turn: reviewer comments are reproduced in full, each followed by our response. Comments are numbered for reference only; the numbering is ours, not the reviewers’.

# Reviewer 1

### R1-01

**Comment.** Aquaculturists in developed countries complain about over-regulation, and trust among producers and regulators can prove lacking. Why is that, and what can be done about it? Focusing on the American Midwest, Hartenstine and Carlton interviewed 34 producers, applying the theory and approach of trust ecology and the producers’ own words to identify issues, infer the drivers of producers’ attitudes, and identify ways for trust to be enhanced going forward. This is a justifiable application of grounded theory, where the subjects’ words are parsed closely so that the interviewees effectively tell the researcher what the real issues are. The downsides of the approach is that the results are qualitative, not given to statistical analysis, and that the applicability of the findings to contexts beyond those of the study may be limited.

**Response.** We thank the reviewer for their summary of the manuscript. We address the question of the transferability of our findings in our responses to R1-02 and R1-38 below.

### R1-02

**Comment.** The latter downside is very much at issue here. Because PLoS One is an international journal, the authors need to make the case that their findings are of interest not only in the U.S. Great Lakes region, but more generally to aquaculture or agriculture. The authors note limitations of the study’s findings to the region — is the key contribution of this study, then, that the methodology that they applied is of general interest, justifying publication of this manuscript in a general, international journal, rather than say, the North American Journal of Aquaculture? I urge the authors to make their case in their revision and their letter of response to review.

**Response.** We believe that this comment is not totally applicable to our paper, since PLOS One explicitly mentions that perceived significance of the research is not a criterion for evaluating manuscripts: “We evaluate research on the basis of scientific validity, strong methodology, and high ethical standards—not perceived significance” (<https://journals.plos.org/plosone/s/journal-information>).

However, we want our work to be widely read and appreciated by an international audience. To that end, we have added some additional connections between US farming and farming in the rest of the world. This included adding references to situate US agriculture as a system whose practices and technologies are exported globally and to establish that reliance on advisors and extension services (relationships that are often dependent on trust) extends well beyond the United States and into the Global South. In addition, we have added detail to the conclusion, connecting trust in our paper to a potential broader agrarian transition.

### R1-03

**Comment.** The role of the Cooperative Extension Services of the respective states in the USA should be explained for international readers. The history and impact of Cooperative Extension will not be known to them.

**Response.** We have clarified cooperative extension’s role in this paragraph and gone into more detail in the methods as requested in comment 15.

### R1-04

**Comment.** The prose is generally good, but can be strengthened with attention to marks that I made on the manuscript. I hope that the authors will take them as constructive.

**Response.** Thank you for these detailed annotations. We have reviewed them and made the appropriate modifications to the manuscript. As a result, it is more precise, logically flows better, and is easier to understand.

### R1-05

**Comment.** At line 27, I suggest a revision so that the authors do not judge the value of their own work.

**Response.** We agree and have removed the evaluative language. The sentence formerly read “These findings present an important case study illustrating how trust influences…” and now reads “These findings illustrate how trust influences…”

### R1-06

**Comment.** At line 58, would “farmers” be more precise that “individuals”?

**Response.** Yes — changed to “farmers,” as suggested.

### R1-07

**Comment.** At line 87, I suggest addition of the question, “How can trust be promoted?”

**Response.** We have made this addition, thank you.

### R1-08

**Comment.** The meaning of the terms “trust richness” and “trust evenness” is not known to most readers, and while they are defined much later, they ought to be given in the Introduction at line 135.

**Response.** We agree and have made this edit, thank you.

### R1-09

**Comment.** The acronym NRM — which I take to be natural resources management — should be spelled out at line 143.

**Response.** We agree and have spelled this out. As the abbreviation was used only once in the manuscript, we have replaced it with “natural resource management” rather than retain an abbreviation, which we hope improves readability for a general audience.

### R1-10

**Comment.** Aquaculture should be defined for as general a readership as that of PLoS One at line 161, perhaps as the controlled production of aquatic organisms.

**Response.** We have added a definition at first substantive use, quoted directly from NOAA: aquaculture is “the breeding, rearing, and harvesting of fish, shellfish, algae, and other organisms in all types of water environments”.

### R1-11

**Comment.** I suggest that the sentence about natural fish stocks at line 163 mention not only that they are a finite resource, but also that they are fully exploited.

**Response.** We have revised the sentence as suggested: it now reads that “wild fish stocks are finite and often fully or over-exploited,” supported by the most recent FAO assessment (FAO 2026) alongside the existing citation.

### R1-12

**Comment.** At line 181, the authors should provide the number of the Executive Order, its date, and if possible, its URL.

**Response.** We have added all three. The text now names Executive Order 13921 of May 7, 2020, “Promoting American Seafood Competitiveness and Economic Growth,” and the reference list gives the Federal Register URL.

### R1-13

**Comment.** I note that the Introduction fails to mention what the trust-related issues are. I suggest that they are permitting, financing, and use of introduced species. My point is that the nature of the issues pertinent to the study region should be given to provide the reader with a greater sense of context.

**Response.** We appreciate this comment and have added some relevant issues to the introduction.

### R1-14

**Comment.** At line 197, I suggest the addition of a few words of clarification, yielding: government aquaculture oversight.

**Response.** Added. The sentence now reads that the interview guide elicited “opinions and comments on trust in government aquaculture oversight, management, and regulation.”

### R1-15

**Comment.** “Extension” as a capitalized entity will not be known to many international readers. The mission of Cooperative Extension Services at American Land Grant Universities should be briefly explained and their impact noted with a supporting citation, perhaps at line 199.

**Response.** We have done so.

### R1-16

**Comment.** At line 206, which Great Lakes States are at issue? They should be specified.

**Response.** Thank you. We have clarified this in the manuscript.

### R1-17

**Comment.** At line 207, the authors correctly note that there is minimal aquaculture in the actual US Great Lakes, but they should add that there is considerable aquaculture activity in the inland waters of states bordering the Great Lakes.

**Response.** We added substantial detail on the aquaculture market in the Great Lakes in this section, including citing numbers from the Census of Aquaculture.

### R1-18

**Comment.** At line 217, the authors mention criteria for producers that were interviewed, but they fail to tell the reader what those criteria were.

**Response.** We moved that specific paragraph to the results in order to address a different comment, but we added a note about inclusion criteria to this part of the methods section.

### R1-19

**Comment.** At line 229, we need a supporting citation or URL for NVivo version 13.

**Response.** We have added a citation and corrected the version designation, which now follows the developer’s recommendation: NVivo 13 (2020, R1).

### R1-20

**Comment.** At line 232, we need a supporting citation for the trust ecology framework. Examples supporting its utility would be nice.

**Response.** We have added a citation as requested. We believe that the revisions to the introduction in response to other comments give more detail on the utility of the framework.

### R1-21

**Comment.** At line 237, we need a supporting citation for Cohen’s kappa coefficient.

**Response.** We have added Cohen (1960) at first mention of the statistic. The interpretation benchmark for the observed value was already supported by Landis and Koch (1977), which we retain.

### R1-22

**Comment.** A word is missing from line 247, perhaps “of”.

**Response.** We’ve fixed it: the sentence now reads “Positive trust is evidence of trust diversity.”

### R1-23

**Comment.** At line 253, what is the SRQR reporting guideline? Do we need a supporting citation?

**Response.** We have spelled the guideline out at first use as the Standards for Reporting Qualitative Research and added the supporting citation (O’Brien et al. 2014). In addressing this comment we also noticed that the checklist was cross-referenced to the wrong supplemental file — the text pointed to S2 Table, which is the dispositional trust table, rather than to S1 Table, which is the SRQR checklist. This has been corrected.

### R1-24

**Comment.** The Results section lacks some key material. The authors should tell us that they interviewed n = 34 aquaculture producers. Perhaps some demographics on age, sex, education, and how long they have been engaged in aquaculture. What were the issues — permitting, inspection, disease, introduced species — on which they engaged with regulatory authorities? This background would provide context for understanding the results.

**Response.** Due to privacy concerns and in keeping with our IRB’s requirements, we are unable to add demographic information to the manuscript. However, we have added a more detailed description of the types of issues that these producers might engage with.

### R1-25

**Comment.** Lines 269-271 define trust richness and trust evenness. These definitions should be copied and pasted into the Introduction at line 135.

**Response.** We have added the definitions as requested.

### R1-26

**Comment.** At lines 273 and 274, I suggest addition of “mention of” for clarity, as I’ve marked on the manuscript itself.

**Response.** Thank you. We have clarified this text during our revision.

### R1-27

**Comment.** In column 2 of Table 1, I suggest that the heading read: Number of interviews presenting evidence. The table should underline subheadings or indent lines to better show its organization.

**Response.** We have adopted the suggested column heading and set the four trust dimensions in italics so that the organization of the table is clear at a glance.

### R1-28

**Comment.** At line 387, the acronym DNR should be defined at first usage.

**Response.** Thanks, we have done this.

### R1-29

**Comment.** At line 430, there is a word missing, perhaps “relations” or “experiences”.

**Response.** Corrected using the reviewer’s suggestion: the sentence now reads “Many producers described experiences with these types of people.”

### R1-30

**Comment.** Lines 438-444 duplicate a passage above, likely due to an errant cut-and-paste, and should be deleted.

**Response.** The duplicated paragraph has been deleted.

### R1-31

**Comment.** At line 458, the acronym FDA should be defined as U.S. Food and Drug Administration at first usage. It will not be known to all international readers.

**Response.** Fixed, thank you.

### R1-32

**Comment.** At line 484, another unmentioned source of procedural inconsistency would be changing oversight policies.

**Response.** We made this addition. Thank you.

### R1-33

**Comment.** At line 497, the acronym DEP should be spelled out at first usage.

**Response.** Fixed, thank you.

### R1-34

**Comment.** At line 550, what does “neutral function” mean?

**Response.** We agree that this line was confusing and have revised the paragraph to clarify our intent: that a lack of trust could serve as a baseline for building trust in the future. The sentence “In our data, lack of trust appeared to serve a neutral function. The instances of lack of trust we identified were mostly workable baselines for building trust…” has been cut down to “These instances of lack of trust were mostly workable baselines for building trust…” We also added “or distrust” to the opening sentence of the section, so that the absence being described is explicitly an absence of both.

### R1-35

**Comment.** At lines 602-603, the authors mention federal grant money or insurance programs similar to those for other form of agriculture. Especially for an international readership, they would do well to mention an example or two, perhaps price supports for soybeans in a bumper-crop year, or disaster relief funds for cotton following flooding.

**Response.** We agree and have named specific programs. The passage now cites the subsidized US federal crop insurance program, price support programs for commodity crops, and the Emergency Assistance for Livestock, Honeybees, and Farm-raised Fish Program (ELAP), noting that ELAP did not cover farm-raised foodfish until after our study period.

### R1-36

**Comment.** At line 619, aquaculture is not an “institution”, it is a “sector”.

**Response.** We have updated this wording.

### R1-37

**Comment.** At line 627, procedural trust is at issue between producers and regulatory AGENCIES, not “processes”.

**Response.** We have updated this language, thank you.

### R1-38

**Comment.** Above in my general comments, I question the fit of this manuscript within a general, international journal. I would suggest that line 737 provides a context to present the case that this methodological approach be applied in other aquacultural or agricultural contexts, that using the producers’ own words to identify issues provides regulatory oversight agencies the opportunity to understand and address trust issues with the sectors that they regulate. That argument would increase the impact of this study.

**Response.** We have added a closing paragraph at the location the reviewer identifies. It situates our findings within the broader challenge of agricultural transition, notes that aquaculture’s friction with regulators stems in large part from its position as an expanding sector governed by an apparatus built around established commodity agriculture, and distinguishes the sector-specific details of our trust-building and trust-repair findings from the broader mechanisms they illustrate — mechanisms relevant wherever a regulated primary sector and its oversight agencies are in tension. We note that transferring those mechanisms to other settings requires attention to local regulatory histories, institutional relationships, and cultural context. Finally, and most directly to the reviewer’s point, we note that by eliciting producers’ perspectives directly, regulatory agencies can better understand trust issues and dynamics in the sectors that they regulate — that is, the approach we take here is itself portable, independent of the specific findings it produced in this case.

# Reviewer 2

### R2-00

**Comment.** This is a well-written and valuable manuscript that applies the trust ecology framework to an important and understudied context. The qualitative approach yields rich insights, and the findings are clearly presented and connected to the framework.

**Response.** We thank the reviewer for this generous assessment of the manuscript, and for the specific and constructive suggestions that follow.

### R2-01

**Comment.** Briefly note the novelty of applying the trust ecology framework to aquaculture to strengthen the abstract’s opening.

**Response.** We have added this to the abstract.

### R2-02

**Comment.** Briefly hint at why stronger relationships and advocacy matter (e.g., for policy experimentation or sustainable growth) to increase impact.

**Response.** We have added some of this detail to the abstract, thank you.

### R2-03

**Comment.** Add a bridging sentence explicitly connecting the challenges of farmer-expert relationships to the trust ecology framework.

**Response.** We have added a bridging sentence to the beginning of the discussion of trust ecology.

### R2-04

**Comment.** Briefly note that the regulatory environment is a known barrier to U.S. aquaculture growth to better set up the research questions.

**Response.** We have added this information to the beginning of that section, thanks.

### R2-05

**Comment.** Integrate the four research questions more smoothly into the final narrative paragraph of the introduction.

**Response.** We believe that the revisions that we made to the introduction in response to this and other comments make the questions flow more naturally.

### R2-06

**Comment.** Briefly explain how you distinguished “positive trust” from “distrust” during coding.

**Response.** We added a paragraph detailing the code development process and inclusion criteria and pointed the reader to the Supplemental Materials for more information. We believe this provides the detail the reviewer was looking for.

### R2-07

**Comment.** Briefly note potential downsides of strong in-group trust in the affinitive trust section (e.g., “us vs. them” perceptions).

**Response.** We have added a couple of sentences to this effect, including a new quote from the transcripts.

### R2-08

**Comment.** Consider briefly linking regulatory inconsistency to effects on innovation or new entrants.

**Response.** We have added a paragraph discussing this issue, which has made this overall section much stronger.

### R2-09

**Comment.** Briefly link each repair strategy in the trust repair section to the trust dimension it targets.

**Response.** We have added several sentences making this connection directly, thank you.

### R2-10

**Comment.** Add slightly more specificity about what “advocacy” might look like in practice (e.g., liaisons, lobbying).

**Response.** We have added a little of this specificity, thank you.

We thank the reviewers again for their time and attention.
